# Omental Well-Differentiated Liposarcoma: US, CT and MR Findings

**Published:** 2009-09

**Authors:** Francesco Meloni, Claudio F Feo, Stefano Profili, Maria Laura Cossu, Giovanni B Meloni

**Affiliations:** 1 *Department of Radiology, University of Sassari, Sassari, Italy*; 2 *Department of Surgery, Istituto di Clinica Chirurgica, University of Sassari, Sassari, Italy*; 3 *Service of Radiology, Paolo Dattori Hospital, Tempio Pausania, Italy*; 4 *Department of Surgery, Chirurgia dell'Obesità, University of Sassari, Sassari, Italy*

**Keywords:** computed tomography, liposarcoma, magnetic resonance, omentum, ultrasound

## Abstract

Liposarcomas are the most common of sarcoma tumours, they are usually located in the lower limbs, retroperitoneum, or abdominal cavity; up to date, only a few cases of omental liposarcoma with different histotype have been described. We present a case of omental well-differentiated liposarcoma and discuss imaging findings on ultrasound, computed tomography, and magnetic resonance to differentiate omental liposarcomas from other abdominal tumour entities.

## INTRODUCTION

Primary tumours of the omentum are very rare, we don't know their incidence and most of the information available is from case reports. Liposarcomas usually involve the limbs, retroperitoneum, or abdominal cavity in adults (1). Up to date, in a review of the English medical literature we found only 14 cases of omental liposarcoma with different histological subtypes.

We present a case of omental well-differentiated liposarcoma and discuss imaging findings on ultrasound (US), computed tomography (CT), and magnetic resonance (MR) scans.

## CASE REPORT

**Figure 1:**
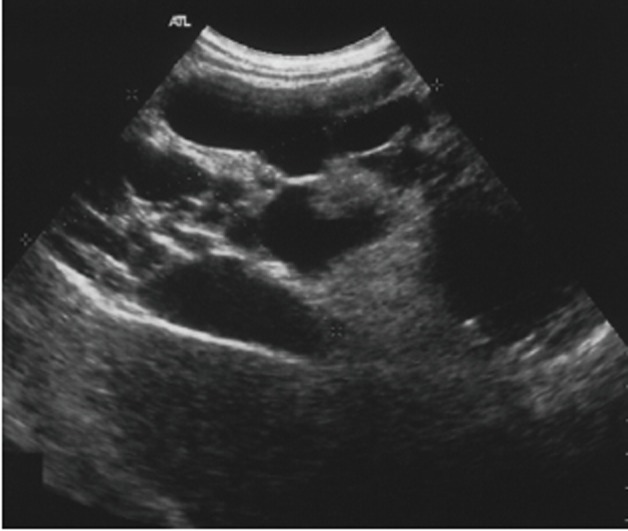
Abdominal US scan revealed a hypomesogastric mass, characterized by two components: a solid portion and a second portion formed by multiple cystoid cavities.

**Figure 2:**
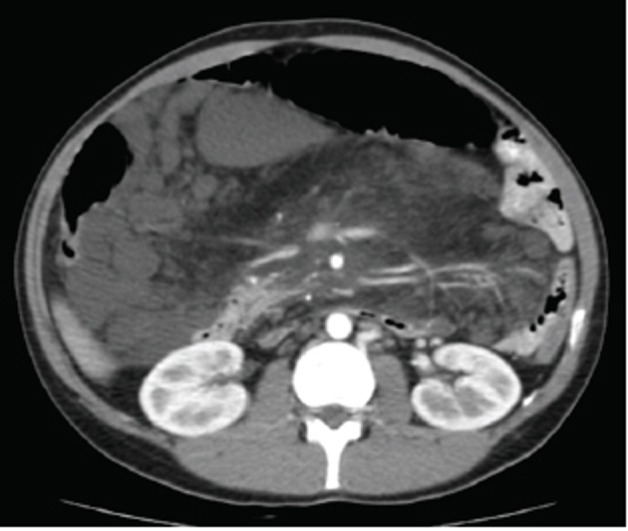
CT scan showed an abdominal mass extended from the hepatic hilus to the pelvis; it was composed of fatty areas (−85HU) near the mesentery and a hyperdense (32HU) portion located dorsally.

**Figure 3:**
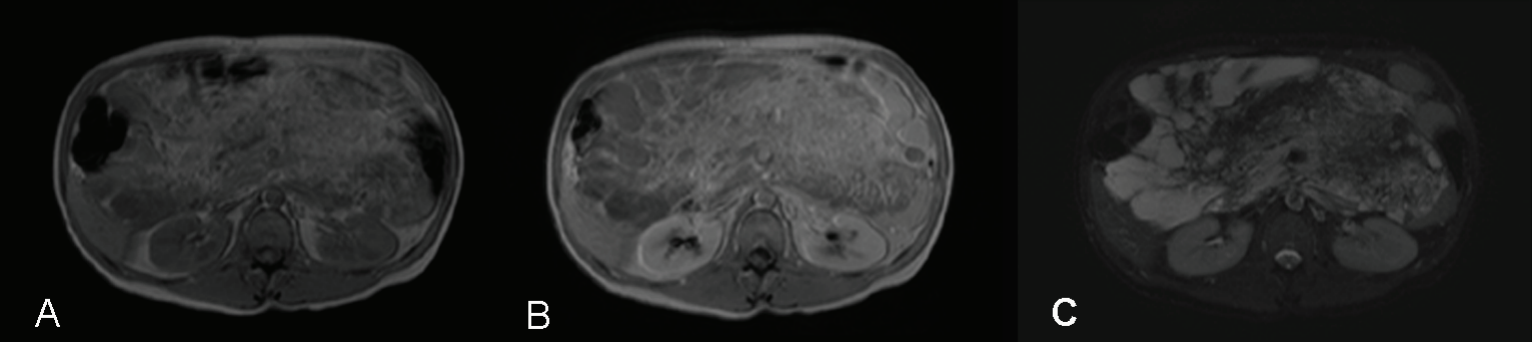
MR examination performed before (A) and after (B) contrast administration with T1- and T2-weighted images and suppression of fatty tissue (C) confirmed an abdominal mass with a dorsally located multicystoid area surrounded by fat.

A 34-year old man suffering from autism presented a 2-year history of worsening abdominal distension. Physical examination revealed a large tender abdominal mass across the midline in the middle and lower quadrants. Laboratory tests were normal. Abdominal US (Philips ATL HDI-5000 system) revealed a hypomesogastric mass, measuring 18 × 13 cm in diameters, characterized by two components: a main hyper-echoic solid portion; and, superiorly, a second portion formed by multiple cystoid cavities (Fig. 1). The mass surrounded the celiac trunk and mesenteric vessels but no flow alterations were detected at duplex and color doppler examination. CT scan (General Electric, High Speed, Single Slice Spiral scanner) showed a 25 × 13 cm abdominal mass extended from the hepatic hilus to the pelvis (Fig. 2). The lesion was adjacent to the great vessels dorsally and to the renal fascia, duodenum, and pancreas laterally; small bowel loops and transverse colon were displaced downward and upward, respectively. It was composed of a fatty area (−85HU) near the mesentery and a hyperdense (32HU) portion located dorsally. MR examination (Shimadzu 1T scanner) performed before and after contrast medium injection with T1-(Fig. 3A, B - TR 243 ms, TE 4 ms) and T2-weighted images and suppression of fatty tissue (Fig. 3C - TR 415 ms, TE 80 ms) confirmed an abdominal mass with a dorsally located multicystoid area surrounded by fat.

The patient underwent surgical excision and pathologic examination revealed an omental well-differentiated liposarcoma. He was discharged after six days and no recurrence was observed during a 5-year clinical follow-up.

## DISCUSSION

Liposarcomas are the most common of sarcoma tumours, they are usually located in the lower limbs, retroperitoneum, or abdominal cavity, and are the most frequent histological type of retroperitoneal tumours (1). Pathologically liposarcomas are malignant tumours formed by lipoblasts and are divided in three subtypes with increasing malignity: well-differentiated liposarcoma, myxoid and round-cell liposarcoma, and pleomorphic (dedifferentiated) liposarcoma (2). Liposarcomas can appear at any age but they occur more frequently in the sixth and seventh decades of life with a male/female ratio of 2:1 (3). These tumours are rarely located in the mesentery, Hasegawa T *et al* have studied 32 cases of dedifferentiated liposarcoma: 27 were situated in the reroperitoneum and 5 in the mesentery (4). Symptoms are usually nonspecific: abdominal discomfort or distension, constipation, and rarely lower limb oedema may be present.

Liposarcomas often contains different histological subtypes with benign and malignant areas in the same lesion, therefore CT and MR scans may vary according to the dishomogenity in tissue composition. Particularly, the signal density on CT and signal intensity on MR images from a well-differentiate liposarcoma are similar to those found in normal fat, and differential diagnosis with other abdominal tumour entities can be difficult.

A well-differentiated liposarcoma is characterized by thinner and more irregular septa than a lipoma; these septa present signal density (CT) and intensity (MR) similar to those found in muscles and may show enhancement by gadolinium-contrast MR imaging. Some authors found focal areas with a signal different from fat tissue both in well-differentiated liposarcomas and benign lipomas (5). These features should be evaluated for the differential diagnosis with more invasive liposarcomas, such as the dedifferentiated subtype characterized by a worsen prognosis and higher recurrence rate (6), and the myxoid subtype that accounts for 50% of all liposarcomas (7). Areas that disappear in the fat suppression sequences and show different enhancement levels on MR scan are consistent with a well-differentiate liposarcoma.

Myxoid liposarcomas show a dishomogeneous aspect with CT attenuation values lower than muscular tissue. Fat and soft tissues distribution pattern may sometimes produce fluid attenuation values, therefore the lesion appears cystic on scans without contrast medium misleading diagnosis. MR images reveals a signal intensity similar to water; however it is sometimes possible to observe indented, linear, or amorphous areas with increased signal intensity on T1- and mean signal intensity on T2-weighted images. These features indicates the presence of intratumoral fat and allow the correct diagnosis. Furthermore, these lesions are better defined after intravenous contrast medium injection showing a slow progressive and reticular enhancement, which indicates the solid nature of the tumour.

The diagnosis of a dedifferentiated liposarcoma should be considered when an abdominal mass shows on CT and MR imaging intense enhancement, adjacent organs invasion, vascular infiltration, calcification or ossification, and areas of necrosis or haemorrhage (8, 9).

In our case, we observed a liposarcoma with two components: a solid area with fat signal intensity and a second multicystoid portion. Fat suppression sequences confirmed the presence of adipose tissue in the former area and the cystic appearance of the latter. No foci with signal intensity different from fat were detected; CT and MR scans didn't show any significant variation after intravenous contrast medium injection. There was no evidence of other organs or vascular invasion. These features were consistent with a well-differentiated lipasarcoma which was then confirmed by pathological examination.

In conclusion, omental well-differentiated liposarcomas are extremely rare and differential diagnosis with other abdominal tumour entities can be challenging. We stress the importance of fat suppression sequences and gadolinium administration on MR scan to identify abdominal masses of difficult diagnosis.
